# Measurement of Ensemble TRPV1 Ion Channel Currents Using Droplet Bilayers

**DOI:** 10.1371/journal.pone.0141366

**Published:** 2015-10-29

**Authors:** Viksita Vijayvergiya, Shiv Acharya, Sidney P. Wilson, Jacob J. Schmidt

**Affiliations:** Department of Bioengineering, University of California Los Angeles, Los Angeles, California, United States of America; University of Newcastle, AUSTRALIA

## Abstract

Electrophysiological characterization of ion channels is useful for elucidation of channel function as well as quantitative assessment of pharmaceutical effects on ion channel conductance. We used droplet bilayers to measure ensemble ion channel currents from membrane preparations made from TRPV1-expressing HEK cells. Conductance measurements showed rectification, activation by acid and capsaicin, and inhibition by capsazepine, SB 452533, and JNJ 17293212. We also quantitatively measured concentration-dependent inhibition of channel conductance through determination of capsazepine IC_50_ in agreement with previously published studies using patch clamp. These results, combined with the reduced apparatus and material requirements of droplet bilayers, indicate that this platform could be used for study of other physiologically relevant ion channels.

## Introduction

Electrical measurement of cellular ion channels using patch clamp can directly probe function at the single channel and ensemble levels.[1] However, the apparatus, time, and training required to perform these measurements can be prohibitive. Electrophysiological measurements using lipid bilayer membranes have decreased apparatus and labor requirements and also allow a greater degree of control over membrane and solution composition.[2–4] Reconstitution of ion channels in lipid bilayers is well established, typically for studies of single channels, although bilayer measurement of ion channel ensembles has also been reported.[5]

One technique for lipid bilayer formation, droplet bilayers, involves aqueous droplets present within an oil solution.[6,7] Lipid molecules contained in the aqueous and/or oil phases self-assemble on the aqueous-oil interfaces to form lipid monolayers; mechanical contact of the droplets unites the monolayers to form a lipid bilayer. Droplet bilayers are particularly amenable to measurement of ion channel ensembles because addition of ion channel-containing vesicles or membrane preparations to the aqueous droplets can result in the formation of bilayers with large numbers of incorporated ion channels and measured currents from pA to tens of nA or more.[8]

As a large number of engineered ion channel-expressing cell lines is available commercially, there is considerable promise in the use of this technique for study of a wide and diverse range of channels. We have used the droplet bilayer technique to study TRPM8 and hERG ion channels, showing modulation of ion channel conductance as a function of voltage, time, temperature, and presence of various drugs as a function of concentration.[9–11] The droplet bilayer technique is also particularly amenable to parallelization and automation;[12–15] this, combined with numerous commercially available engineered ion channel-expressing cell lines, gives this technology considerable potential for drug discovery and screening assays.

Here we report measurements of the TRPV1 ion channels using droplet bilayers. TRPV1 (transient receptor potential vanilloid-1) is a non-specific cation channel that can be activated by multiple stimuli, such as heat, voltage, acid, and various chemicals such as capsaicin.[16,17] We made membrane preparations from engineered TRPV1-expressing HEK cells and electrically studied these membrane preparations using a droplet bilayer platform. Measurement of the TRPV1 membrane preparations with droplet bilayers showed currents with magnitude ranging from single channel (~10 pA) to multiple channel ensembles (> 1000 pA) that exhibited voltage-dependent conductance and rectification similar to patch clamp.[18,19] The measured currents were also activated by capsaicin and acid and inhibited by SB 452533, JNJ 17293212, and capsazepine. Concentration-dependent inhibition of TRPV1 acid-activated currents by capsazepine were also observed. Measurement of these ensemble currents and chemical conductance modulation, coupled with the small amount of material required and decreased apparatus and expertise requirements, make this approach attractive for studies of physiologically relevant ion channels.

## Materials and Methods

1,2-diphytanoyl-sn-glycero-3-phosphocholine (DPhPC) was obtained from Avanti Polar Lipids. Capsaicin, phenylmethylsulfonyl fluoride (PMSF) and all commonly used chemicals were purchased from Sigma-Aldrich. SB 452533, JNJ 17293212, and capsazepine were purchased from Tocris Biosciences. Human embryonic kidney (HEK) cells expressing TRPV1 were provided by Librede Inc. For the aqueous solutions used for droplet bilayer measurement, the buffer 150 mM NaCl, 10 mM HEPES and 5 mM CaCl_2_, pH 7.4 (buffer Na150) was used.

### Cellular membrane preparations

Two plates of cells (~20 million cells) expressing TRPV1 (provided by Librede Inc.) were collected into 7 mL of aqueous buffer containing 150 mM KCl, 50 mM HEPES pH 7.4, and 1 mM PMSF and were sonicated (Branson Digital Sonifier) at 20 percent amplitude for two seconds, repeating three times to lyse the cells. This solution was centrifuged at 5000 g for 5 min and the supernatant was centrifuged at 100,000 g for 1 hr at 4°C to collect the pellet consisting of mostly membrane vesicles[20]. The pellet was re-suspended in the same buffer and homogenized using a dounce homogenizer ten times and aliquoted into 30 μL volumes for use or storage at -80°C. The total protein concentration was characterized to be 2–4 mg/ml using a Bradford protein assay. The membrane preparations were stored at -80°C and used within one week by diluting them into buffer Na150 at ratios of 1:100 to 1:10000 before use.

### Formation of lipid bilayers and conductance measurements

**Fig 1:**
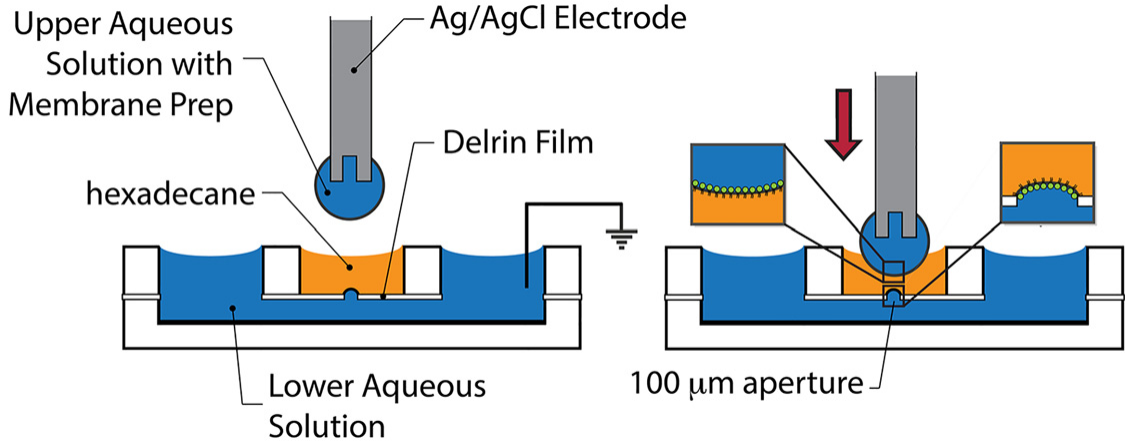
Droplet bilayer schematic. (Left) Side wells are loaded with the lower aqueous solution. The central well is then loaded with hexadecane containing 10 mg/ml DPhPC. The lower aqueous solution and center well are separated by a 75 μm thick Delrin film containing a 100 μm diameter aperture. An aqueous droplet containing membrane preparation is suspended from the center Ag/AgCl electrode, which is lowered into the center well from above. (Right) Lipid monolayers spontaneously form at the aqueous/hexadecane interfaces (insets). Further lowering of the electrode contacts the monolayers to form a lipid bilayer which is bounded by the Delrin aperture.

Droplet bilayer formation used the same apparatus and methods as previously described[10,11] (Fig 1). Briefly, the lower chamber was filled with measurement buffer, followed by addition of 80 μL of DPhPC in hexadecane (10 mg/ml) to the middle chamber. A droplet of solution containing measurement buffer and diluted membrane preparation was placed on the end of a Ag/AgCl electrode mounted on a micromanipulator and lowered into the hexadecane solution in the center well. A waiting time of 30 minutes allowed for formation of lipid monolayers on the hexadecane/aqueous interfaces of the lower aqueous solution and the droplet. The droplet was lowered further to bring the two monolayers into contact, with the contact area bounded by a 100 μm diameter circular aperture in a 75 μm thick Delrin sheet (McMaster-Carr). Bilayer area was estimated from measurement of capacitive currents resulting from the application of a 10 mV amplitude 8 Hz triangle wave. Experiments were performed at room temperature (23°C).

Currents were recorded with an Axopatch 200B integrating patch clamp amplifier (Axon Instruments). Signals were filtered at 1 kHz with a low pass Bessel filter and digitized at 5 kHz with Digidata 1322A (Axon Instruments). pClamp9 software (Axon Instruments) was used to generate voltage clamp commands, acquire membrane currents, and analyze digitized data. For analysis, multichannel currents are reported without leak subtraction or capacitance compensation. Measurements of ion currents in presence of agonist or antagonist compounds were done by adding aliquots of solutions containing them to the lower chamber in the same buffer.

## Results and Discussion

Droplet bilayers have been explored for sensing and screening applications with microfluidic devices, arrays, and automation hardware.[6,12–15,21,22] Since measurement of ion channel ensemble currents have been reported for a number of ion channels in droplet bilayers,[8–11] these technological capabilities may enable electrophysiological screening of pharmaceuticals. DPhPC is a commonly used artificial lipid in droplet bilayer applications, as it is much less labile than unsaturated lipids. One of the aims of this study was to assess the compatibility of droplet bilayers using DPhPC to measure single channel and ensemble currents of TRPV1.

**Fig 2:**
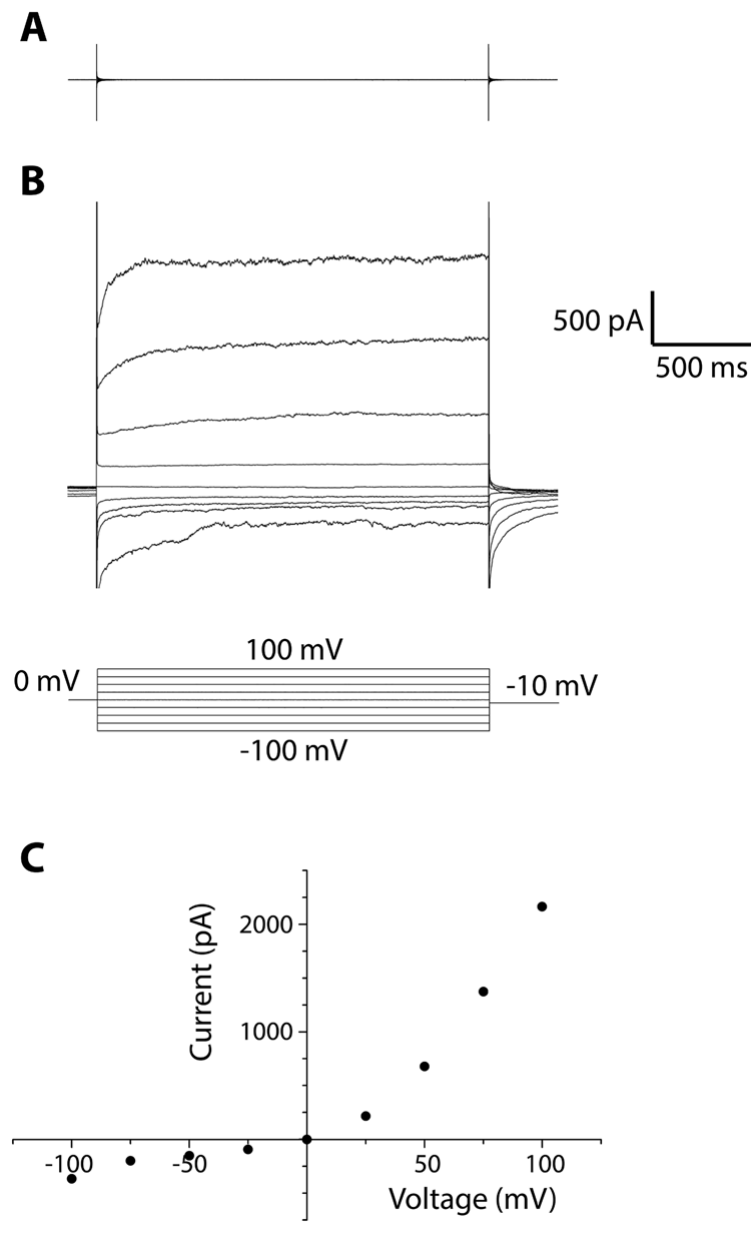
Measurement of ensemble currents from TRPV1 membrane preparations in Na150 buffer. Rectifying currents for control HEK cells (A) and TRPV1 expressing HEK cells (B) were measured during application of 0 mV for 400 msec followed by voltage steps to -100 mV to +100 mV for 2 sec. (C) Measured currents from (B) between 0.5–2.2 sec were averaged and plotted versus voltage.

Large currents, hundreds to thousands of pA in magnitude, were observed that were not observed in control measurements of membrane preparations made from HEK cells not expressing TRPV1 channels (Fig 2, Representative data, N = 10). Rectifying currents were observed during application of constant trans-membrane potentials stepping from -100 mV to +100 mV. This rectification is consistent with measurements of TRPV1 expressed in HEK cells[18], as well as measurements of TRPV1 channels reconstituted into Giant Unilamellar Vesicles (GUVs).[19] The observation of rectifying currents measured with identical buffer on both side of the membrane suggests that there is a net orientation of the incorporated ion channels in this measurement, which was also noted by Cao et al.[19]

**Fig 3:**
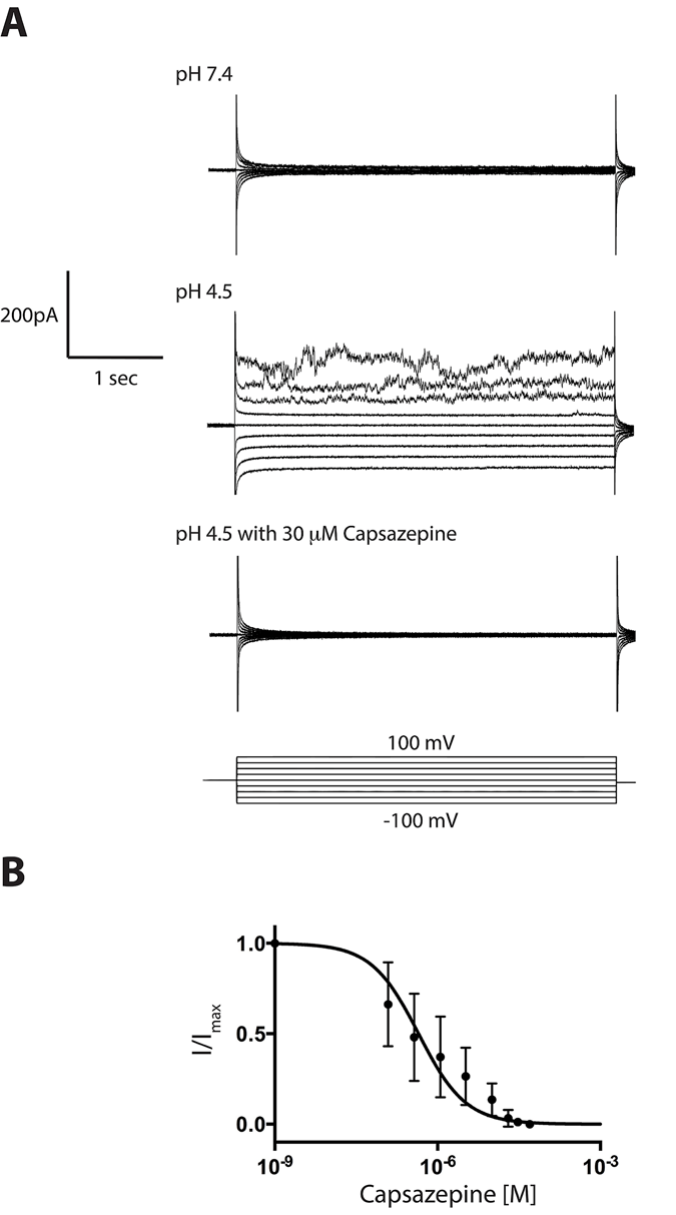
Activation by acid and inhibition by capsazepine. A: (Top) Measurement of TRPV1 membrane preparations at pH 7.4 showed little current, but acidification of the lower solution to pH 4.5 increased the measured current for positive applied voltage (Middle). Following addition of 30 μM capsazepine to the lower (pH 4.5) solution, the currents were completely blocked (Bottom). For these traces, the applied voltage was stepped from -100 mV to +100 mV for 2 sec following 400 msec at 0 mV as shown. B: Repetition of this experiment but with increasing concentrations of capsazepine showed concentration-dependent inhibition of the measured currents. The current measured at 100 mV was normalized to the value before capsazepine addition and plotted versus capsazepine concentration. This plot was fit to a simple binding curve and the IC_50_ value from this fit was 446 ± 112 nM (N = 2).

To test for pH sensitivity of our TRPV1 membrane preparation we measured TRPV1 membrane preparations in the presence of Na150 buffer (pH 7.4) on both sides of the bilayer. After the addition HCl to the lower chamber to obtain a solution pH of 4.5, the measured conductance was observed to increase and exhibit rectification, indicating that the acid was activating the TRPV1 channels, as seen in previously reported measurements of TRPV1 reconstituted in GUVs[19]. Addition of capsazepine to the bottom chamber to a final concentration of 30 μM blocked the measured currents entirely (Fig 3A). The observed rectification may have resulted from a large net orientation of the incorporated channels with their extracellular sides facing the lower aqueous solution for acid activation, or a mixture of both orientations may be present in the membrane, with only the orientation with extracellular sides facing the lower aqueous solution able to be acid-activated.

In a subsequent experiment, we repeated the pH 4.5 activation of TRPV1 conductance and serially added capsazepine to the lower solution, to obtain capsazepine concentrations of 41 nM, 123 nM, 370 nM, 1.13 μM, 3.3 μM, 10 μM, 20 μM, and 30 μM while measuring the concentration-dependent reduction of these pH activated currents. The current at each concentration was allowed to stabilize for ~30 seconds. Currents were then measured at +100 mV, averaged over 3 seconds, and normalized to the current measured before capsazepine addition and plotted versus capsazepine concentration (Fig 3B). Fit of this graph to a simple binding curve gave an IC_50_ value of 446 ± 112 nM (N = 2), which is larger than previous reported IC_50_ value of 58 nM measured from currents activated by pH 5.5.[23]

**Fig 4:**
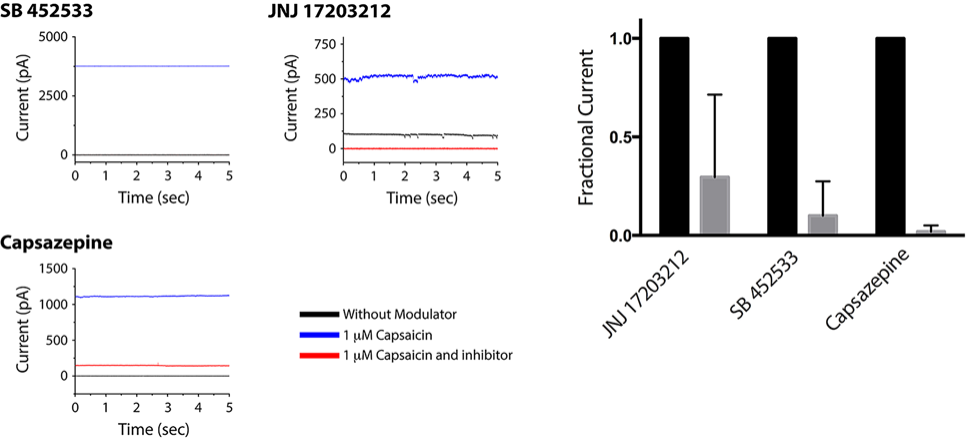
Inhibition of capsaicin-activated TRPV1 currents. (Left) Bilayers made from TRPV1 membrane preparations were measured before compound addition (gray traces), after addition of 1 μM capsaicin (blue traces) andafter addition of blockers (red traces). The blockers tested were 1 μM SB 452533, 1 μM JNJ 17293212, and 700 nM capsazepine. For these traces, the applied voltage was +60 mV. (Right) The current measured in the presence of capsaicin (black) and after addition of the blockers (gray) was normalized by the capsaicin current to show average reduction over several experiments (N = 3, SB 452533 and capsazepine; N = 2 JNJ 17293212).

We also measured the ability of capsaicin-competitive TRPV1 blockers SB 452533[24], JNJ 17293212[25], and capsazepine[26] to inhibit capsaicin-activated TRPV1 currents. With the aqueous solutions consisting of Na150 and TRPV1 membrane preparations, following bilayer formation an aliquot of capsaicin was added to the lower chamber to obtain a final concentration of 1 μM. The bilayer current was monitored during application of +60 mV and, after the increased current stabilized, SB 452533 and JNJ 17293212 were added to a final concentration of 1 μM, and capsazepine was added to a final concentration of 700 nM in separate experiments (Fig 4). In each of the three experiments, the current amplitude decreased and stabilized near 0 pA.

Using a droplet bilayer platform, we have measured large currents from membrane preparations of TRPV1-expressing HEK cells. Measurements of TRPV1 showed voltage rectification, acid and capsaicin activation, and pharmaceutical inhibition of the conductance in agreement with previously reported studies using patch clamp. The availability of a large number of ion channel-expressing cell lines, combined with the ease of sample preparation, the small amount of material required, and the relative simplicity of bilayer measurements compared to patch clamp, make this an attractive technique for ion channel characterization and assessment of pharmaceutical and environmental modulators of ion channel activity. The compatibility of droplet bilayers with automation and array technologies also give this platform considerable potential for screening applications.
